# Risk factors for severe *Chlamydia pneumoniae* pneumonia in children: a retrospective case–control study

**DOI:** 10.3389/fped.2026.1834503

**Published:** 2026-06-03

**Authors:** Di Hu, Yingqian Zhang, Ran Ma, Chunxiao Ba, Peijuan Wang

**Affiliations:** 1 Graduate School, Hebei Medical University, Shijiazhuang, Hebei, China; 2 Department No. 3 of Respiratory Medicine, Hebei Children’s Hospital, Shijiazhuang, Hebei, China; 3 Hebei Clinical Medicine Research Center for Children’s Health and Diseases, Shijiazhuang, Hebei, China; 4 Hebei Key Laboratory of Pediatric Allergy and Immunology, Hebei Children's Hospital, Shijiazhuang, Hebei, China

**Keywords:** children, *Chlamydia pneumoniae* pneumonia, immunoglobulin a, risk factors, severe pneumonia

## Abstract

**Objective:**

This study aimed to investigate the clinical characteristics of *Chlamydia pneumoniae* pneumonia (CPP) in children and analyze the risk factors for progression to severe pneumonia.

**Methods:**

A total of 133 children with CPP were retrospectively enrolled from Hebei Children's Hospital between 2023 and 2025. Disease severity was classified according to the Guidelines for the Management of Community-Acquired Pneumonia in Children (2024 revision), and patients were divided into a non-severe group (*n* = 99) and a severe group (*n* = 34). Demographic characteristics, clinical manifestations, and laboratory findings were compared between groups. Multivariate binary logistic regression analysis was performed to identify independent factors associated with severe disease.

**Results:**

Compared with the non-severe group, children in the severe group had a higher peak temperature and longer duration of fever (both *P* < 0.001). No significant differences were observed in the proportion of mixed infections or the distribution of pathogens between the two groups (all *P* > 0.05). Laboratory findings showed a lower lymphocyte percentage and higher levels of WBC, CRP, and IgA in the severe group (all *P* < 0.05). Multivariate analysis identified decreased lymphocyte percentage (OR = 0.943, 95% CI 0.895–0.994) and elevated IgA (OR = 2.227, 95% CI 1.284–3.972) as factors associated with severe CPP.

**Conclusion:**

Decreased lymphocyte percentage and elevated serum IgA are associated with severe CPP in children, suggesting potential roles in risk stratification. Further multicenter prospective studies are warranted to validate and confirm these findings.

## 1 Introduction

Community-acquired pneumonia (CAP) is an acute lower respiratory tract infection. It remains one of the leading causes of morbidity and mortality among children worldwide (1). *Chlamydia pneumoniae* (CP) is an important etiological agent of pediatric CAP. It accounts for approximately 1%–2% of cases (2). CP infection is predominantly community-acquired and is known to spread in clusters in high-density settings such as schools. Although infection can occur across all pediatric age groups, preschool- and school-aged children are considered more susceptible (3). Epidemiologically, CP demonstrates a clear seasonal pattern, with higher incidence typically observed during autumn and winter, In addition, prior studies have suggested that its circulation follows a cyclical epidemic pattern (4, 5).

In Europe, PCR-based surveillance data indicate a marked rise in CP infections in recent years. In southeastern France, CP qPCR positivity increased approximately 19-fold between 2018 and 2024 (6). In southern Germany, positivity rates rose from approximately 0.3% during 2015–2020 to 2.6% in 2024, with localized peaks exceeding 6.0% by late 2024 (7). Comparable trends have been observed in Asia, where seropositivity rose from 4.3% in 2022 to 5.9% in 2023 (8).

The incubation period of CP infection is usually 3–4 weeks. Most cases present with mild and self-limited respiratory symptoms and have a favorable prognosis (9). However, atypical clinical manifestations and slow disease progression often make etiological diagnosis difficult. This may delay appropriate treatment and lead to lung injury (10). In some children, particularly those with impaired immune function, severe infection may occur. Rare cases may even result in death (11).

Consequently, detecting risk factors for severe *Chlamydia pneumoniae* pneumonia (CPP) at an early stage is essential. This research seeks to evaluate the clinical features of CPP in children and the drivers of disease severity. Improving early detection will support timely clinical management and help prevent acute lung injury and chronic pulmonary sequelae.

## 2 Materials and methods

### 2.1 Study population

A total of 133 children aged 1 month to 16 years with *Chlamydia pneumoniae* pneumonia (CPP) were retrospectively enrolled. All patients were hospitalized at Hebei Children's Hospital between 2023 and 2025.

Inclusion and exclusion criteria:

Children were included if they met all of the following criteria:

1. Diagnosis of *Chlamydia pneumoniae* pneumonia (CPP) according to the Guidelines for the Management of Community-Acquired Pneumonia in Children (2024 revision). Severity classification was defined independently according to the same guideline. Severe pneumonia was diagnosed when any of the following criteria were present:
2. poor general condition, refusal to feed, or signs of dehydration;
3. altered consciousness;
4. cyanosis or dyspnea;
5. markedly increased respiratory rate (≥70 breaths/min in infants or ≥50 breaths/min in older children);
6. oxygen saturation ≤0.92 at sea level or <0.90 at high altitude;
7. radiographic findings showing multilobar involvement or involvement of ≥2/3 of a single lobe;
8. pleural effusion;
9. extrapulmonary complications (12).
10. Etiological testing: Sputum samples were collected from 133 children at admission. Respiratory pathogens were detected using a polymerase chain reaction (PCR) respiratory pathogen detection kit (Ningbo Haiershi Gene Technology Co., Ltd., China).
11. Availability of complete clinical data.

Exclusion criteria included:

1. presence of underlying diseases, such as congenital lung dysplasia, congenital heart disease, or moderate-to-severe chronic respiratory diseases (e.g., uncontrolled asthma).
2. history of immunosuppressive therapy or known immunodeficiency.
3. recent use of systemic antibiotics prior to admission (within 48 h before hospitalization).
4. incomplete clinical records.
5. discharge against medical advice after hospitalization.

Written informed consent was obtained from the legal guardians of all participants.The study was approved by the Medical Ethics Committee of Hebei Children's Hospital (Approval No. 2025034-46).

### 2.2 Data collection

Clinical data were obtained from the hospital electronic medical record system. The collected information included demographic characteristics, clinical manifestations, physical examination findings, laboratory results, and imaging findings.

### 2.3 Statistical analysis

Statistical analyses were performed using SPSS software (version 27.0; IBM Corp., Armonk, NY, USA). Normality and homogeneity of variance were assessed before analysis. Continuous variables with normal distribution and equal variance were expressed as mean ± standard deviation. Differences between groups were compared using the independent-samples t-test. Continuous variables that were not normally distributed were expressed as median (interquartile range) [M (P25, P75)]. The Mann–Whitney U test was used for comparisons between groups.Categorical variables were expressed as percentages. Group differences were analyzed using the *χ^2^*test or Fisher's exact test.Univariate and multivariable logistic regression were performed to identify potential influencing factors.

## 3 Results

### 3.1 Comparison of general characteristics and clinical features

A total of 133 children diagnosed with CPP were included in this study, including 99 cases in the non-severe group and 34 cases in the severe group. No significant differences were observed between the two groups in sex distribution, age, or body weight (all *P* > 0.05). Similarly, there were no significant differences in total disease duration, cough characteristics, length of hospitalization, presence of coinfection, or organ function indicators (all *P* > 0.05) (Table 1).

**Table 1:** The demographic and clinical characteristics.

Variables	Mild group	Severe group	*t*/*Z*/*χ^2^*-value	*p*-value
Gender (M/F)	70/29	22/12	0.427	0.513
Age (years)	11.00 (9.80,13.00)	11.00 (9.00,13.00)	−0.286	0.775
Weight (kg)	49.74 ± 2.09	47.98 ± 2.60	0.525	0.601
Peak temperature (℃)	37.50 (36.80,38.40)	38.90 (38.37,39.22)	−5.394	<0.001
Duration of fever (d)	1.00 (0.00,4.00)	5.50 (1.00,7.00)	−3.681	<0.001
Duration of hospitalization (d)	7.00 (5.00,9.00)	7.00 (6.75,9.00)	−1.689	0.091
Total duration of illness (d)	12.00 (9.00,16.00)	14.50 (9.75,18.00)	−1.825	0.068
Cough days (d)	9.00 (7.00,14.00)	9.50 (7.00,15.00)	−0.344	0.731
Cough (*n*,%)	98 (99%)	33 (97.1%)	0.637	0.447
Dyspnea (*n*,%)	9 (9.1%)	1 (2.9%)	1.376	0.451
Mixed infections (*n*,%)	72 (72.7%)	28 (82.4%)	1.281	0.258
Liver dysfunction (*n*,%)	21 (21.2%)	8 (23.5%)	0.080	0.778
Renal dysfunction (*n*,%)	7 (7.1%)	3 (5.9%)	0.057	0.812

Data are presented as mean ± standard deviation, *n* (%), or median (interquartile range); Liver function panel: total bilirubin (TBil), direct bilirubin (DBil), alanine aminotransferase (ALT), aspartate aminotransferase (AST), total bile acids (TBA), and gamma-glutamyl transferase (GGT); Renal function panel: urea (Urea), creatinine (Cr), and uric acid (UA).

However, compared with the non-severe group, the severe group exhibited significantly higher peak body temperature [38.90 (38.37,39.22) °C vs. 37.50 (36.80,38.40) °C] and longer duration of fever [5.50 (1.00,7.00) days vs. 1.00 (0.00,4.00) days] (both *P* < 0.001) (Table 1).

### 3.2 Analysis of mixed pathogen infections

**Figure 1:**
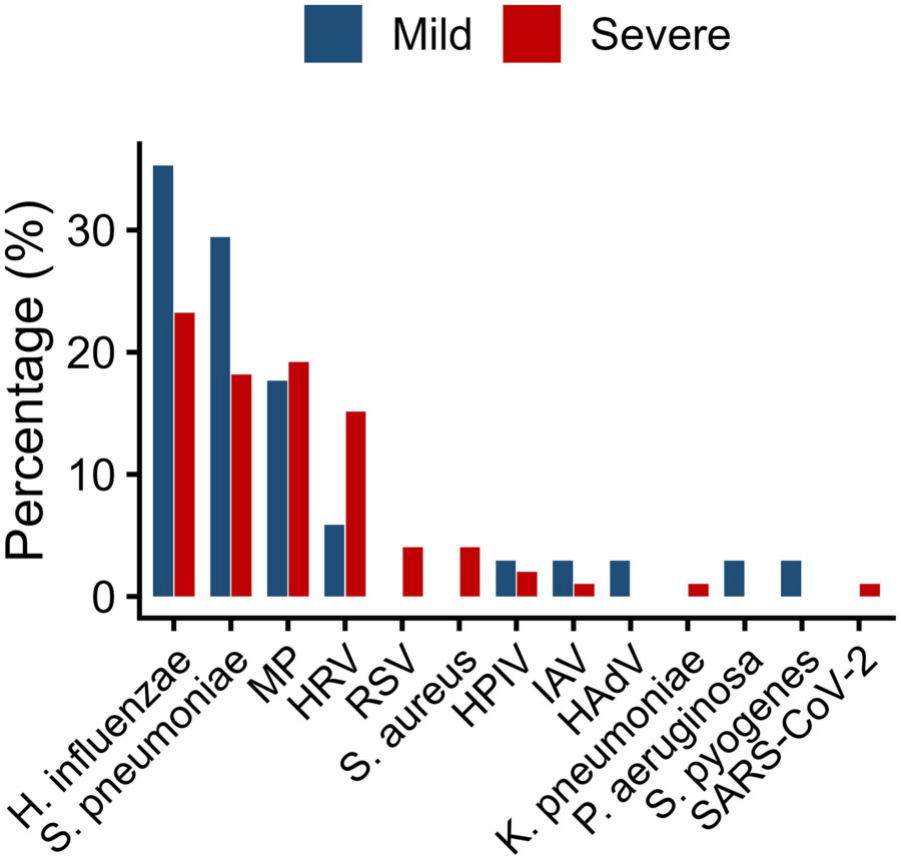
Distribution of respiratory pathogens identified among the study population. The horizontal bar chart illustrates the frequency and percentage of the 13 pathogens detected in the subjects.HAdV, human adenovirus; H. influenzae, *Haemophilus influenzae*; HPIV, human parainfluenza virus; HRV, human rhinovirus; IAV, influenza A virus; K. pneumoniae, Klebsiella pneumoniae; MP, *Mycoplasma pneumoniae*; P. aeruginosa, *Pseudomonas aeruginosa*; RSV, respiratory syncytial virus; S. aureus, *Staphylococcus aureus*; S. pneumoniae, *Streptococcus pneumoniae*; S. pyogenes, *Streptococcus pyogenes*; SARS-CoV-2, severe acute respiratory syndrome coronavirus 2.

Mixed pathogen infections were observed in both groups.In the non-severe group, the most common coinfecting pathogens were *Haemophilus influenzae* [23 cases (23.2%)], *Mycoplasma pneumoniae* [19 cases (19.2%)], *Streptococcus pneumoniae* [18 cases (18.2%)], and rhinovirus [15 cases (15.2%)]. In the severe group, *Haemophilus influenzae* [12 cases (35.3%)] and *Streptococcus pneumoniae* [10 cases (29.4%)] were the most frequently detected pathogens (Figure 1).

No significant differences were showed in the distribution of *Mycoplasma pneumoniae*, *Streptococcus pneumoniae*, or *Haemophilus influenzae* between the two groups (*χ^2^* = 0.04, 1.92, and 1.899, respectively; all *P* > 0.05). Other pathogens, including rhinovirus, respiratory syncytial virus, parainfluenza virus, influenza A virus, severe acute respiratory syndrome coronavirus 2, adenovirus, *Streptococcus pyogenes*, *Klebsiella pneumoniae*, *Pseudomonas aeruginosa*, and *Staphylococcus aureus*, also showed no significant differences between the two groups (all *P* > 0.05, Fisher's exact test) (Table 2).

**Table 2:** Etiology of mixed infections.

Variables	Mild group n(%)	Severe group n(%)	*χ^2^*-value	*p*-value
MP	19 (19.2%)	6 (17.6%)	0.04	0.842
HRV	15 (15.2%)	2 (5.9%)		0.236
RSV	4 (4.0%)	0 (0.0%)		0.572
HPIV	2 (2.0%)	1 (2.9%)		1.000
IAV	1 (1.0%)	1 (2.9%)		0.447
SARS-CoV-2	1 (1.0%)	0 (0.0%)		1.000
HAdV	0 (0.0%)	1 (2.9%)		0.256
S. pneumoniae	18 (18.2%)	10 (29.4%)	1.92	0.166
S. pyogenes	0 (0.0%)	1 (2.9%)		0.256
H. influenzae	23 (23.2%)	12 (35.3%)	1.899	0.168
K. pneumoniae	1 (1.0%)	0 (0.0%)		1.000
P. aeruginosa	0 (0.0%)	1 (2.9%)		0.256
S.aureus	4 (4.0%)	0 (0.0%)		0.572

Data were analyzed using the *χ^2^* test or Fisher's exact test.

### 3.3 Comparison of laboratory parameters

No significant differences were observed between the two groups in neutrophil percentage, platelet count, hemoglobin, D-dimer, fibrinogen, IgM, or IgG levels (all *P* > 0.05) (Table 3).

**Table 3:** Laboratory indicators.

Variables	Mild group	Severe group	*t/Z*-value	*p*-value
N (%)	60.96 ± 9.27	64.81 ± 13.21	−1.531	0.133
L (%)	29.29 ± 7.73	24.84 ± 10.62	2.129	0.040
PLT ( × 10^9^/L)	328.67 ± 90.97	324.58 ± 95.82	0.216	0.830
WBC ( × 10^9^/L)	8.85 (7.30,10.70)	10.07 (7.45,12.81)	−2.044	0.041
CRP (mg/L)	5.00 (2.02,14.80)	10.20 (2.83,40.82)	−2.232	0.026
Hb (g/L)	133.00 (126.00,137.00)	131.00 (126.00,139.50)	−0.704	0.481
D-dimer (mg/L)	0.26 (0.19,0.37)	0.27 (0.22.0.39)	−1.313	0.189
FIB (g/L)	3.23 (2.77,3.66)	3.60 (2.82,4.38)	−1.209	0.227
IgA (g/L)	1.40 (1.02,1.90)	1.67 (1.39,2.21)	−2.865	0.004
IgM (g/L)	1.44 (1.13,1.82)	1.19 (0.82,1.62)	−1.038	0.299
IgG (g/L)	11.13 (9.72,12.59)	11.39 (9.46,12.36)	−0.859	0.390

Data are presented as mean ± standard deviation, *n* (%), or median (interquartile range).

N%, neutrophil percentage; L%, lymphocyte percentage; PLT, platelet count; WBC, white blood cell count; CRP, C-reactive protein; Hb, hemoglobin; FIB, fibrinogen; IgA, immunoglobulin A; IgM, immunoglobulin M; IgG, immunoglobulin G.

Compared with the non-severe group, the severe group showed a significantly lower lymphocyte percentage [(24.84 ± 10.62)% vs. (29.29 ± 7.73)%], higher white blood cell count [10.07 (7.45,12.81) × 10⁹/L vs. 8.85 (7.30,10.70) × 10⁹/L], and higher C-reactive protein levels [10.20 (2.83,40.82) mg/L vs. 5.00 (2.02,14.80) mg/L] (all *P* < 0.05) (Table 3).

Serum IgA levels were also significantly higher in the severe group compared with the non-severe group [1.67 (1.39,2.21) g/L vs. 1.40 (1.02,1.90) g/L] (*P* = 0.004) (Table 3).

### 3.4 Risk factor analysis for severe CPP

Univariate analysis identified WBC, CRP, lymphocyte percentage (L%), and IgA as variables associated with severe CPP in children (all *P* < 0.05) (Table 4).

**Table 4:** Univariate logistic regression analysis of severe CPP.

Variables	*β*	SE	Wald *χ^2^*	*P*	OR	95%CI
WBC (× 10^9^/L)	0.168	0.071	5.654	0.017	1.183	1.030–1.359
L (%)	−0.062	0.026	5.820	0.016	0.940	0.893–0.988
CRP (mg/L)	0.031	0.010	9.034	0.003	1.032	1.011–1.053
IgA (g/L)	0.834	0.285	8.559	0.003	2.302	1.317–4.025

WBC, white blood cell count; L%, lymphocyte percentage; CRP, C-reactive protein; IgA, immunoglobulin A.

To minimize overfitting and consider clinical relevance, L% and IgA were included in the final multivariable logistic regression model. Multivariable analysis demonstrated that decreased L% and elevated IgA were independently associated with severe disease (both *P* < 0.05) (Table 5).

**Table 5:** Multivariate logistic regression analysis of severe CPP.

Variables	*β*	SE	Wald *χ^2^*	*P*	OR	95%CI
L (%)	−0.059	0.027	4.844	0.028	0.943	0.895–0.994
IgA (g/L)	0.800	0.295	7.384	0.007	2.227	1.284–3.972

L%, lymphocyte percentage; IgA, immunoglobulin A.

## 4 Discussion

*Chlamydia pneumoniae* (CP) is an obligate intracellular pathogen with a unique biphasic life cycle consisting of elementary bodies and reticulate bodies (13). Its replication and persistent infection depend on the intracellular environment of host cells. CP infection can also induce complex immune and inflammatory responses (14).

Recent global disease burden data indicate that lower respiratory tract infections remain a major cause of death in children. In recent years, infections caused by atypical pathogens, including CP, have shown an increasing trend, particularly in the post-pandemic period (15–17). CP infection is usually mild or self-limited. It is often characterized by hoarseness and persistent dry cough (18). However, in some children, the disease may progress to severe pneumonia. It may also lead to extrapulmonary complications and is associated with the development or exacerbation of chronic asthma (19, 20). Therefore, identifying factors associated with severe disease is important for clinical risk stratification.

Our single-center retrospective case–control study systematically analyzed the clinical characteristics of *Chlamydia pneumoniae* pneumonia (CPP) in children and explored factors associated with severe disease. The results showed that children in the severe group had higher peak temperature and longer duration of fever, suggesting a more pronounced inflammatory response during disease progression. In laboratory findings, the severe group showed a lower lymphocyte percentage and higher levels of white blood cell count, C-reactive protein, and IgA. In univariate analysis, WBC, CRP, L%, and IgA were all associated with severe disease. Considering clinical relevance and model stability given the relatively limited number of severe cases, lymphocyte percentage and IgA were selected for inclusion in the multivariable logistic regression model. Multivariable logistic regression analysis showed that decreased lymphocyte percentage and elevated IgA were associated with severe CPP, suggesting that these immune-related markers may be linked to disease severity.

### 4.1 Mixed infection and severity of CPP

In the present study, a relatively high proportion of mixed infections was observed in both the non-severe and severe groups (72.7% vs. 82.4%), which is consistent with previous reports demonstrating that CP infections are frequently accompanied by co-infecting pathogens (21). Although the proportion of mixed infections was higher in the severe group, the difference did not reach statistical significance. Regarding specific pathogens, *Haemophilus influenzae* and *Streptococcus pneumoniae* were the most commonly identified co-infecting bacteria in both groups, with higher detection rates in the severe group; however, no statistically significant differences were observed. Based on these findings, mixed infection was not included in subsequent univariate and multivariate logistic regression analyses.

A previous study on *Chlamydia trachomatis* pneumonia suggested that mixed infection may play a role in the development of severe disease (22); however, no comparable association was identified in the present study of CPP. A case report by Mărginean et al. further suggested that CP infection may be accompanied by complex immune regulatory changes that could, to some extent, influence disease progression (23). These findings suggest that, beyond pathogen burden, host immune responses may also be involved in the progression of CPP. Accordingly, further validation in larger multicenter studies is needed.

### 4.2 Decreased lymphocyte percentage and the risk of severe CPP

Lymphocytes play an important role in immune defense and inflammatory regulation during respiratory infections in children. Previous studies have reported that lymphopenia in community-acquired pneumonia is associated with an increased risk of severe disease and higher mortality, and that persistent lymphopenia tends to be linked to worse outcomes compared with transient decreases (24, 25). As an obligate intracellular pathogen, CP relies on the host immune response for infection control, and prior studies have suggested that impaired immunity may allow CP to persist within host cells and sustain inflammatory responses (26, 27). In the present study, the lymphocyte percentage was significantly lower in children with severe CPP than in those with non-severe disease, and this association remained significant in the multivariate analysis (OR = 0.943, 95% CI: 0.895–0.994). The decreased lymphocyte percentage observed in severe CPP may reflect a relatively impaired adaptive immune response during infection, which may be associated with an increased risk of severe disease.

### 4.3 Elevated IgA and the severity of CPP

Immunoglobulin A (IgA) is the most abundantly produced immunoglobulin in the human body. It plays an important role in mucosal immune defense and systemic immune regulation (28). In the present study, elevated serum IgA levels were significantly associated with the risk of severe CPP (OR = 2.227).

This finding is partly consistent with previous studies on community-acquired pneumonia in adults. One study reported that serum IgA levels were significantly higher in non-survivors than in survivors among patients with CAP, suggesting a potential association between elevated IgA levels and poor clinical outcomes (29). Although the study populations and pathogens differed, these findings collectively indicate that increased IgA levels during respiratory infections may not necessarily reflect effective immune protection. Instead, they may indicate stronger immune activation or a higher inflammatory burden.

From an immunological perspective, the function of IgA is closely related to its structural form and receptor signaling. Monomeric serum IgA can interact with Fc*α*RI and induce inhibitory signaling, thereby suppressing inflammatory responses in neutrophils and monocytes. In contrast, polymeric IgA or IgA-containing immune complexes can trigger activating signals through Fc*α*RI. This process promotes the release of inflammatory mediators and enhances cellular effector functions. Under conditions of severe infection or persistent antigenic stimulation, IgA is more likely to form immune complexes (30). In this context, these immune complexes may enhance the pulmonary inflammatory cascade and may be associated with the severe clinical manifestations observed in our study.

Previous clinical studies have reported that *Chlamydia pneumoniae* infection can trigger IgA vasculitis as well as various skin and mucosal manifestations. These findings suggest that CP infection may act as a trigger for abnormal IgA-mediated immune responses. Such observations provide indirect evidence that IgA-related immune mechanisms may participate in inflammatory injury during CP infection (31–34).

It should be noted that experimental studies in animal models suggest a potential protective role of IgA in the later stages of infection. Neonatal animals with IgA deficiency were more likely to develop pulmonary dysfunction and structural lung injury following CP infection, although pathogen clearance was only minimally affected (35).

Therefore, the role of IgA in infectious diseases is not purely protective. Its effects may vary depending on its molecular form and the immune microenvironment. During the acute inflammatory phase, elevated IgA levels may amplify inflammatory responses. During the recovery phase, IgA may help limit excessive inflammation and promote tissue repair. The association between elevated IgA and severe disease observed in this study may therefore reflect its pro-inflammatory role during the acute stage of infection.

## 5 Conclusion

In summary, decreased lymphocyte percentage and elevated serum IgA levels were associated with severe Chlamydia pneumoniae pneumonia in children, suggesting that they may reflect disease severity. In clinical practice, these markers may provide some reference for assessing the severity of Chlamydia pneumoniae pneumonia.

However, our study has several limitations. First, this was a retrospective, single-center study conducted at Hebei Children's Hospital, which may introduce selection bias and limit the generalizability of the findings to other populations or regions. Second, due to financial constraints of patients during their hospital visits, some laboratory examinations were incomplete. This resulted in limited clinical data and a relatively small sample size in the severe group, which may reduce the statistical power of certain analyses. Third, our study focused solely on serum IgA levels without assessing local IgA in the airway mucosa. Therefore, the exact role of IgA during the acute inflammatory phase of Chlamydia pneumoniae infection remains to be further elucidated. In the future, we will focus on IgA-mediated mucosal immune mechanisms and conduct multicenter, prospective studies to validate our findings and clarify the clinical value of these factors across different populations and regions.

## Data Availability

The original contributions presented in the study are included in the article/Supplementary Material, further inquiries can be directed to the corresponding author.
